# M. Dupuytren and Mad. Boivin on Fibrous Tumors and Polypi of the Uterus

**Published:** 1835-01-01

**Authors:** 


### ON FIBROUS TUMORS, OR POLYPI OF THE UTERUS.

1. DES TUMEURS FIBRO-CELLULEUSES DE L'UTERUS; VULGAIREMENT DESIGNÉES SOUS LE NOM DE POLYPES DE LA MATRICE.

[*Leçons Orales de M. Dupuytren, Article* XVII.]

II. ON PEDICULATED FIBROUS TUMORS OR POLYPI.

[*Treatise on Diseases of the Uterus and its Appendages. By Mad. Boivin and A. Dugès. Translated by G. O. Heming, F.L.S. &c. Octavo, pp.* 559.]

POLYPI of the uterus have attracted some attention in the last few years, and have been illustrated by the labours of the best accoucheurs and morbid anatomists of the day. M. Dupuytren has paid considerable attention to the subject, or rather to that part of it in which the surgeon is peculiarly interested. The affliction which weighs on this eminent, we might almost say illustrious man, and threatens with his loss humanity and science, surrounds his writings with a fresh and indeed with a painful charm, and confers on them something approaching to the character of posthumous productions. Should his present illness lead to the event anticipated by all, more particularly by himself, his native country will have reason to deplore the deprivation of an ornament to his profession and to her. But we quit with haste the unpleasing theme.

We shall not attempt to amalgamate the separate articles before us, but set before our readers in succession the facts and the opinions of Mons.

Dupuytren, and those of the editors of the treatise on diseases of the uterus. We shall not, of course, extract or allude to the elementary matter in either, supposing, as we think we may safely do, that this is familiar to the greater portion of our readers.

The Baron commences his lecture with the details of two cases in which a polypus existed, and was removed by the operation of division of its pedicle. The cases give rise to some remarks, and the latter lead to a general history of fibrous tumors of the uterus, drawn from a vast fund of observation and experience. We shall start with this general account of the disease.

Fibrous tumors, says the Baron, though various in form, are generally rounded, and composed of a tissue resembling that of ligaments and tendons. They are found in all parts where the fibrous element abounds, but more frequently in the uterus than in any other organ. It is important to ascertain and to remember the situations from which they spring.

1. Some arise from the external surface of the uterus, between its proper tissue and its peritoneal tunic by a peduncle which sometimes is extremely small; this and some cellular tissue are the sole connexion between the polypus and fibrous structure of the uterus. The tumor thus formed projects into the abdomen, lifts up the peritoneum, and may attain but trifling or very large dimensions.

2. Others form in the actual substance of the uterus, equidistant from its inner and its outer surface. Yet they do not appear to grow from that structure, but are merely placed in it, separating its fibres, and sometimes so insulated as to seem encysted. They present no peduncle. Their increase is slow, and though usually smaller than the previous variety, they occasionally equal the head of an adult in their dimensions. In some instances, they augment equally in every direction; in others, they project towards the cavity of the uterus, or towards that of the peritoneum. These tumors are beyond the operations of surgery.

3. This appears to be an intermediate and unnecessary class. It consists of such, with peduncles or without them, as spring rather from the outer or the inner surface of the uterus. In the latter case they approach the first kind: in the former they will be found to resemble the next.

4. These tumors are often situate on the internal surface of the uterus, and constitute either simple protuberances into the uterine cavity, or arise, which is more frequent, by distinct peduncles.

The pedunculated tumors, springing from the internal surface of the uterus are those which are usually known as polypi, and consist of a root, a peduncle or neck, and a larger portion or a body. Their surface is covered by a fine adherent membrane, continuous with the mucous tunic of the uterus, by which it appears to be formed.

It is difficult, in many instances, to discover by the touch the peduncle of the polypus, unless it be extremely long, and the polypus itself has passed into the vagina. The root of the tumor is that portion which is actually planted in the uterus, and by which the supply of vessels and of nourishment is carried. It consists of arteries, veins, lymphatics, cellular and fibrous tissue. This circumstance should be remembered, for the tendency occasionally shewn to the reproduction of these tumors after their removal by an operation, is most probably owing to some remains of the morbid growth. Thus, recapitulates M. Dupuytren, polypi may or may not have

D 2

a peduncle, and this is generally dependent on the situation from which they sprout.. Those which are formed in the parietes of the uterus, and those which proceed from its external surface, or indeed from its internal, provided that they receive and exhibit an investment from its fibrous texture, are deficient in a peduncle. The others, in which this part is presented, have been adequately noticed. The latter are usually known under the name of fibrous polypi; the former have been designated fibrous tumors of the uterus.*

The length of the peduncles is extremely variable; some being barely distinguishable from the tumor, and others being two or three inches long. The greater the polypus the greater is usually the elongation of the peduncle, whilst the latter becomes generally thinner as it lengthens. In an instance related by M. Dupuytren the peduncle had become so weak that it spontaneously broke. This is not a frequent accident, for powerful tractions are generally borne, in consequence of the resistance of the fibrous tissue. When the peduncle is soft and thin simple torsion will detach it, but M. Dupuytren prefers division, as less likely to leave portions of the growth behind.

The peduncle is composed of arteries, veins, lymphatics, cellular tissue, and probably of nerves. The existence of arteries, sometimes of large size, might appear to forbid, or, at least to form an argument against division. Yet the reasonable fear is dispelled by experience, and hæmorrhage is found to be extremely rare. M. Dupuytren has many times divided the peduncles of polypi in which the vessels were of considerable size, without the occurrence of hæmorrhage. The presence of lymphatics is frequently obvious, commonly to be inferred. But the nerves, if they exist, are small, and the tumor is possessed of trivial sensibility.

The size which the tumor attains is influenced, no doubt, by the age and the vigour of the patient; but it is more affected by the quantum of compression exerted by the uterus. The polypi of greatest magnitude are such as are implanted in the external surface of the uterus, and grow into the abdomen where little resistance is presented to them. Under any circumstances, great differences are noticed in the dimensions of polypi, some being as small as a grain of millet, and others weighing fifteen to thirty pounds. The largest which M. Dupuytren has witnessed was twenty-five pounds in weight, but the elder M. Gaulthier de Clanbry has published an instance in which the tumor weighed thirty-nine pounds, and had acquired the vast size of thirty-five inches and a quarter in its vertical circumference, and twenty-nine inches and a quarter in its horizontal.

Previous to the occurrence of organic alterations, polypi are usually whitish, smooth, and similar in appearance to the surface of the healthy uterus. When inflamed, they become reddened, and brownish or greyish when sloughing spontaneously, or from the application of the ligature. Their consistence is in general remarkable, equal indeed to that of the intervertebral fibro-cartilages. M. Dupuytren suspended a weight of several hundred pounds to some large polypi, without occasioning their laceration. They are often so elastic, as to rebound when thrown upon the ground.

---

* In this country they are commonly termed "fleshy tubercles." The nomenclature is inferior.—*Eds.*

Their form presents varieties. They are usually globular or ovoid, not unfrequently angular or uneven on the surface, and, when of any magnitude, almost always lobulated externally by fissures. M. Dupuytren has seen some resembling in figure mushrooms reversed, and others conical, with the smaller end below. Trivial as they may appear, it is useful to become acquainted with the forms that polypi assume. As an instance of the necessity of examining carefully the form of a tumor in the vagina, the lecturer relates a case in which an able surgeon pronounced the disease to be cancer. M. Dupuytren ascertained by examination, that the tumor was globular below, and surmounted by a peduncle above. He performed the operation and cured the patient.

M. Dupuytren shews that the polypus is covered by a prolongation of the mucous membrane lining the interior of the uterus. Like mucous membrane, it inflames, becomes congested, gives rise to muco-purulent discharges, and is subjected to ulceration.

Ulcers on the cervix of the uterus, and especially on the os tincæ, are frequently observed, and not unfrequently mistaken for ulcerated scirrhus. The two affections are distinguished by the redness, the irregularly rounded form, and the whitish bottom of the simple ulcer.

M. Dupuytren passes in review once more the textures and divisions that compose the polypus. We need not recapitulate the student's lesson. Yet one or two points deserve consideration. Thus, M. Dupuytren alludes to the opinion, indeed the declaration, that the peduncle exists in such polypi only as have cleared the constricted cervix of the uterus, and expanded uncompressed in the vagina. The peduncle is made to depend on pressure exerted on the portion of the tumor next the uterus. The study of facts disproves the speculation; for pedunculated polypi are found quite inclosed in the uterine cavity, and are seen to originate from different portions of the exterior of the cervix, and even from the outer surface of the uterus itself. No constriction on the stalk can be exercised in these instances.

The elements which mainly compose the polypus are the fibrous and the cellular, the latter much condensed. They are often disposed in equal ratios, but more frequently the one or the other predominates, and confers a character on the succeeding alterations. If the fibrous element is in excess, the polypus, says our author, either does not degenerate at all, or, if at length it does so, assumes an osseous form in lieu of a scirrhous one. If, however, the cellular tissue abounds, the degeneration that ensues is carcinoma; a tendency to which is constant and inevitable after a certain length of time. M. Dupuytren appears to think that inflammation forms the stage, that determines the transition to malignant alteration. We give these opinions as we find them. Perhaps they may admit of question; certainly they should not be unhesitatingly received. We do not indulge in criticism or in argument, as we wish to put our readers in possession of the facts and the opinions of the lecturer.

Whatever be the cause, the cartilaginous or osseous alteration is extremely rare; the cancerous too frequent. Out of one hundred cases, three or four examples of the former transformation are all, perhaps, that will be met with. M. Dupuytren concludes by the assertion, that not only is the existence of the cellular element operative in occasioning scirrhous change of structure, but that serum or serosity combined with the solid textures confers an additional tendency to the degeneration. The serosity, when free in the tumor, is less noxious. We repeat that we present these statements as we find them.

M. Dupuytren having disposed of the influence of tissue proceeds to remark that inflammation is another cause of alterations in the tumor, and even of malignant action. If the polypus projects into the abdominal, or rather into the pelvic cavity, peritonitis and all its consequences may ensue. If the tumor is internal, and invested by the mucous membrane, metritis, and various discharges may result. In both instances malignant transformation may occur. The lecturer here insists on a distinction. In spontaneous malignant action, depending upon tissue, the change commences in the centre, and extends to the circumference; but the cancerous alteration that follows, inflammation begins on the circumference and passes inwards. This is also the case with the ossific alterations.

Cavities are sometimes found in the interior of the fibro-cellular tumors of the uterus. They may be either connate with the tumor itself, or consecutive in their appearance and dependent on softening or morbid alteration of its substance. Saviard and Boudon each relate a case of the former description. In 1823, the surgeons of the Hôpital Saint Louis removed a tumor the size of a child's head, which had hung for some years at the vulva of a female; on division, it presented a cavity in its interior, and resembled the uterus so closely as to lead to the opinion that that organ had been extirpated. The patient died, and the suspicion proved to be erroneous; for the uterus was untouched, and the tumor had been merely an enormous polypus. M. Dupuytren also operated upon one which contained in its centre a considerable cavity. When present, its interior may be smooth and polished, or present projecting fibrous fasciculi, similar to the columnæ carneæ of the heart. Such are the cavities originally formed in fibrous tumors.

But those which result from changes in their constitution, such as softening or degeneration, are filled with a sanious, puriform, or bloody fluid. A surgeon having applied the forceps to a very large fibrous tumor, in order to drag it down in the vagina, broke into a cavity contained in the polypus, and evacuated some dark and excessively offensive matter. The same surgeon removed a polypus from two other patients. In one tumor there were several cavities filled with clots of blood; in the other were three abscesses, containing dark matter.

The reporters term the preceding facts important and "precious," and indulge in an assertion more expressive, we conceive, of their laudable anxiety for the fame of their patron, than of the sober soundness of their judgment. They affirm that it is certain that the fibrous, cancerous, fungoid, fibro-cartilaginous, osseous, and stony growths, formerly considered distinct alterations, are in reality no other than different degrees of the same disease. And on this assumption they build the pleasing but infirm belief, that in all these instances an early operation will prevent the return of the complaint. It is generally thought, perhaps admitted, that morbid growths, not malignant in their origin, may, in process of time, and from various causes, assume a malignant disposition. But the bold idea that many kinds of morbid alteration, some malignant and some not so, have a common and a non-malignant stock, is calculated to awake the scepticism of the cautious.

The exact reasoner asks for proofs, and will not be contented with a chain of reasoning, which, however ingenious, is manifestly inconclusive. M. Dupuytren affects to argue rigorously, he constantly appeals to facts, and his lectures wear a syllogistic habit. Yet it cannot be concealed that, in this instance, he has ventured on some positive yet doubtful affirmations, without attempting substantial proofs.

The succeeding doctrine is equally startling and still more dubious than the last. It is this.

If the tumors are removed at an early period, before "degeneration" commences, a return of the disease is seldom noticed; but if, on the contrary, "degeneration" has begun, a recurrence may be apprehended. So far so good; but the rationale offered by the Baron is debateable. He says that, in the first instance, the investing tissue continuous with and a production of the mucous membrane of the uterus, confines within its limits the disease, and operates as a barrier against its extension to the surrounding textures. When degeneration has attacked the tumor, the investing tissue is itself affected, and the morbid action is disseminated throughout the neighbouring parts. Whilst the morbid growth is circumscribed and thus encysted, its total removal is not difficult. But when it has spread to the surrounding parts, its satisfactory extirpation is impossible.

When we look back on the preceding statements, we discover some bold and magisterial assumptions, unsupported by any thing like reasonable proof. It is an assumption to state that the various morbid growths and morbid alterations of structure of the uterus have a common origin in fibro-cellular productions; it is an assumption to state, or even to imply, that the disease is not cancerous so long as its investing tunic is entire, or, being cancerous, that it does not, so long, contaminate the system. We will not further argue the question than by merely stating that these are assumptions, not merely devoid of proof, but of probability. It appears to us that M. Dupuytren has unphilosophically confounded many diseases of the uterus; and that his cancerous degeneration of fibro-cellular tumors is a mere misnomer of several malignant diseases of the uterus, which never in any stage approached the description or character of polypi. We proceed to another subject—the frequency of fibro-cellular tumors of the uterus.

They were formerly thought to be uncommon. Levret was occupied for seven years in collecting three cases to which he might apply his mode of treatment. A Dutch professor who had never seen an instance of the malady, denied that his country-women were liable to be affected with it. The disease, in point of fact, is frequent. Bayle calculates that, out of every fifty women above the age of thirty-five, one has fibro-cellular tumors of the uterus; such, at least, was the proportion of cases that occurred to him. Portal, in 1770, recorded or obtained a larger proportion. Of twenty uteri which he examined, thirteen contained polypi in their interior. M. Dupuytren believes that the uterus of women advanced in life is very seldom free from them.

M. Dupuytren considers with reluctance and quits with dispatch the causes which give rise to these productions. Those causes are obscure and indeterminate. But it is certain that persons between the ages of forty and fifty are those who most frequently present them. The influence of celibacy or sterility is doubtful.

The reporters refer to sixty-two cases, of which they have collected and analysed the particulars. The points which they attempt to illustrate are the age at which the disease appears—the influence of marriage—the effects of sterility—and the condition of the menstruation.

A. It is manifestly difficult to ascertain the age at which a polypus commences. The nearest approach to the truth which can be made, is, probably, to determine the period at which the symptoms were developed. In five of the sixty-two cases collected, this period is not mentioned. Of the fifty-seven which remain, they appeared—

| | |
|---|---|
| In 1 patient between | 15 and 20 years. |
| 10.............. | 20 and 29 |
| 19.............. | 30 and 39 |
| 23.............. | 40 and 49 |
| 3.............. | 50 and 59 |
| 1.............. | 60 and upwards. |

Thus in the majority the disease was manifested between the age of 40 and 50; whilst 30 to 40 furnished the second proportion. A closer consideration of the individual cases shewed that from 35 to 45 or 48 was the period of life most prone to the complaint.

B. The action of marriage and results of celibacy are interesting, indeed important. Marriage must in this case be viewed through the glass of the philosopher, rather than that of the divine; for, physiologically, those who have enjoyed the act of coition must be looked upon as married.

Of the 62 patients, the chastity or otherwise of four is not recorded.

Of the other 58, 54 were, medically speaking, married.

And, four were single and considered chaste.

C. The determination of chastity is difficult. The fact of sterility, though not always to be ascertained, may generally be so. Of the 62 patients, four, being chaste, may of course be set aside; and seven exhibit no satisfactory evidence upon the subject. The gross number is therefore reduced to 51. Of these there were—

| | | |
|---|---|---|
| Wives having had 1 to 10 children ...... | 39 | 42 |
| Unmarried girls with children .......... | 3 | |
| Wives with no children .............. | 8 | 9 |
| Girls, not maids, without children ...... | 1 | |

Of the wives who had borne children, the greater number had had more than 3, many more than 5, and some so many as 7, 8, and 10.

D. The condition of the menstrual secretion is to be examined. Of the 62 patients—

41 were regular up to the development of the disease.

5 were not regular for several years prior to the first symptoms.

6 had never been regular at any time.

1, aged 48, had been regular but had suffered under leucorrhœa from the age of 18. She had borne, notwithstanding, 6 children.

9 afford no information on the subject.

The reporters well observe that the condition of the menstruation proves little; for irregular catamenia are more likely to depend on polypus or on its cause, than the latter are to arise from *it*.

This careful induction from recorded facts, an induction that may be in some respects fallacious, because those facts may not be strictly accurate, is scarcely illustrated by the brief remarks of Dugès and Boivin. Their section on the predisposing causes of polypi is concise, indeed imperfect. They attribute much to the lymphatic temperament—something to the catamenia.

" We have already remarked," they say, " that the lymphatic temperament is probably one of the principal predisposing causes of albuginous or fibrous tumors of the uterus; we may make the same observation with respect to polypi; but we have sometimes observed a coincidence, worthy of remark, between the existence of polypus attended with profuse discharge by the vagina, and cancer of the mammæ, the liver, or even the face. Perhaps this affection operated only like other debilitating causes, under the influence of which pediculated tumors of the uterus arise. We have, in fact, observed that this disease attacks principally the weak, those who live in low, damp places, and persons of sedentary habits; that it has been preceded by leucorrhœa, and that the catamenia had appeared early and in abundance. In several instances, this flow had been accompanied with membraniform growths; or abortions had taken place, attended with difficult and slow expulsion of the fœtal appendages: all which circumstances might bring on, or manifest, congestion, and habitual, or frequently recurring, inflammatory state of the uterus. It is also at the age and under the particular circumstances in which this congestion usually occurs, that these excrescences appear; seldom observed in the case of the very young,* and still less in the unmarried,† they are equally rare after the cessation of the catamenia, and they are most commonly discovered in cases in which parturition has taken place one or more times, though their origin cannot generally be attributed to any decided local cause." 199.

It must be owned that the causes of polypi of the uterus are concealed in impenetrable darkness. The investigations of pathology have failed, and the study of facts has but slightly contributed to elucidate the subject. The faint and glimmering light of observation amounts, it would seem, to little more than this—that the married and the aged are more subject to the malady than virginity and youth; in other words, that employment and excitement of the organ predispose to the formation of the morbid growth. Unsatisfactory as this small amount of knowledge must be, it is as great as that which we possess in the instance of scirrhus, of fatty tumor, or of other morbid alterations of structure.

On the symptoms of polypus of the uterus we will not dwell, as the common works on midwifery contain the information presented by our authors. We will content ourselves with the remark, that the symptoms vary in some degree with three various situations of the polypus;—within the uterus—at its neck, which is dilating—and in the vagina, after the cervix has dilated. But the experience and the injunctions of M. Dupuytren, on the mode and the results of examination with the finger, are not undeserving of attention.

---

* Pfaff describes a case of polypus which occurred in a female child of two years old. The ligature was applied thrice; and thrice the tumour returned; it was eventually and permanently removed by the forceps; the operation was followed by retention of urine and swelling of the abdomen, which soon subsided.—Richter, Bib. Chir. v. vi., p. 538.—Tr."

† " Siebold has observed three existing at the same time in the case of a person in whom the hymen was perfect.—(*Simson*, p. 22.)"

That examination is available in the two latter stages of polypus—when presenting at the uterine orifice, or actually in the vagina.

When the polypus appears at the uterine orifice, the finger distinguishes a rounded, smooth tumor, usually firm, yet of variable consistence. It is not always easy to decide whether this be a polypus, or thickening of the cervix of the uterus, or, supposing its polypoid nature determined, whether it arises from the border, or the interior of the cervix, or the cavity of the uterus itself. If the cervix is not sufficiently dilated to permit the introduction of the finger, a mistake is easily committed.

*Case.* A young lady, æt. 22, came to Paris from the Provinces, in order to consult two eminent physicians, on account of an uterine disorder. They looked on it as thickening of the uterus, and treated her for that complaint for the period of two months. A celebrated surgeon, M. Dupuytren we suppose, was then consulted. He was told that a remarkable symptom existed—the occurrence of labour-pains every day at the same hour. This awakened his suspicions of the existence of some tumor in the cavity of the uterus. He examined with the finger whilst the patient reclined; but without a satisfactory result. He repeated the examination when she was erect; the os uteri dilated to the size of a franc piece, and the fore-finger passed into it discovered a firm substance, which appeared to be continuous with the parietes of the cervix. The result was still unsatisfactory. But on a subsequent occasion, the surgeon thrust on the finger with some force, and, with difficulty, penetrated into the interior of the uterus. He recognized a large and circumscribed tumor, and pronounced on the existence of a polypus. The diagnosis was ineffectual, for peritonitis carried off the patient in a few days after its announcement. The case was proved to be one of fibrous polypus.

If one examination is unequal to the task of determining the nature of the tumor at the os uteri, several should be practised, and the surgeon should attempt to introduce the finger within the cervix, to circumscribe the tumor, and even to ascertain its seat. M. Dupuytren recommends us to make the attempt, but he does not recommend the gentleness and caution which every prudent surgeon would practise, in order to avoid such fatal consequences as occurred in the case we have just related. The tumor may adhere from inflammation to the sides of the os uteri—or the tumor may grow from the parietes of this or of the uterus itself; in either case the diagnosis is difficult, and the fact should always be remembered. When the polypus springs from the free margin of the os uteri, the remainder of that margin is felt unoccupied, and the cavity of the cervix is not filled.

In the third stage of the tumor, when it has descended into the vagina, it is easily recognized, if of moderate size. If it fills the vaginal cavity, the recognition is more difficult. The finger should, of course, attempt to pass round the polypus and above its body to the neck. If the latter, or constricted portion, is felt to be surrounded by the cervix of the uterus, the polypus evidently issues from that organ; if the orifice is open, and one of its lips continuous with the pedicle, this demonstrates that the polypus grows from the os uteri.

The tumor from its form, or from its bulk, may prevent the finger from reaching its pedicle. When this is the case, M. Dupuytren recommends the adoption of Levret's advice—to seize the tumor with the forceps and drag it down to the vulva; performing at the same time the diagnosis and the operation. A case is related, in which the tumor was so large as to prevent an examination of the ordinary kind from arriving at its pedicle. The following method succeeded. The surgeon placed himself on the left side of the patient, and introduced the forefinger of the left hand between the right side of the tumor and the vagina; ascertaining by this means that the right side of the cervix uteri was free. He repeated this process with the opposite hand for the opposite side, and arrived at a knowledge of the relations of the polypus. M. Dupuytren found that he was only capable of succeeding in this manner. We may observe that, in performing the operation, it was necessary to divide to the extent of half an inch the posterior commissure of the perineum.

When the tumor has cleared the vulva, the diagnosis is generally easy. Yet sometimes it is difficult to determine from what part the pedicle springs, or where the uterus begins. A case in point is detailed by the Baron, who notwithstanding operated with success.

The speculum is useful when the polypus is small. By means of it, M. Dupuytren detected the existence of a great number of small, red, vascular polypi, clustered like a bunch of grapes, and filling the cervix uteri, in a female aged 30, whose case had been mistaken by many medical men.

It must be owned, however, that the diagnosis of polypi is sometimes very difficult, and that errors are not unfrequently committed by experienced men. A case of this description is related. The memory of most surgeons and physicians will probably present such cases to their minds. We should mention a circumstance important to the practical man. Polypi sometimes pass through the uterine orifice suddenly, during the action of the bowels for example. I have known, says Dr. Gooch, several instances in which patients, after this action, have been suddenly seized with retention of urine, and, on examination, a polypus was found in the vagina compressing the urethra. Independently of the possible suddenness of its descent, the surgeon must recollect that a polypus *does* descend—that at one time it is in the uterus, and subsequently in the vagina. What we mean is this:—that a surgeon may examine a patient carefully to-day and discover no polypus, whilst another practitioner examining the same female, in a few days or a week, may find it in the vagina. If the general symptoms of polypus are present, the prudent surgeon will guard his reputation by informing his patient, that although no tumor can be ascertained now, it may be felt at some future time. We lately heard of an instance of this sort. A surgeon attended a lady for symptoms resembling those of polypus. But he could find none, and, concluding that malignant disease of the uterus existed, he cautioned her against hoping any thing from an operation. Soon afterwards she consulted another gentleman, who discovered a polypus in the vagina, operated, and was successful. The first surgeon was wrong, perhaps from ignorance, perhaps from negligence. But his ignorance might have been veiled, had he prudently remembered, and dexterously hinted the circumstance we have alluded to.

Dugés and Boivin advert to the rare occurrence of sanguineous polypi, which become gorged with blood at the epoch of the catamenia. The translator appends a short note to the passage in which this is mentioned. A case of this kind, he says, is given in the Bib. Medicale, v. xxxix. p. 235.

The volume of the tumor was very variable; when it was gorged with blood, it descended beyond the orifice of the vagina, and was seen externally; but the loss of blood which the patient experienced each time caused it to return, and then it could only be perceived by the finger. It was in this state that it was extirpated, without any hæmorrhagy.

To revert to the lectures of M. Dupuytren. He proceeds to describe the symptoms of those fibrous tumors which are formed in the uterine parietes —of those which arise from its peritoneal surface—and, finally, of those which spring from without and around the cervix. We shall only attend to the last.

They are developed around the neck, and in the substance of the tissues encircling the uterus—they are seldom insulated, but usually numerous—are extremely common—are met with in front, behind, or on the sides of the cervix, between the vagina and the rectum, or between the vagina and urethra. The symptoms are determined by the situation, and, from the same cause, are peculiar and characteristic. In the majority of instances, an operation is impossible, but, where possible and proper, it should be performed. Many cases are detailed; we shall instance some.

*Case.* A lady consulted M. Dupuytren for general indisposition, pains in the vagina, but more especially for barrenness. He found, on examination, the cervix uteri surrounded by a sort of circle of prominent and contiguous tubercles, of a fibrous character. Suspecting the co-existence of others, he prosecuted his examination, and found a polypus situated higher on the body of the uterus.

*Case.* M. Dupuytren was consulted by the wife of a confrère. She had been examined by many medical men, who suspected many complaints, but not the right one. After several trials, Mr. Dupuytren detected a tumor on the right side of the cervix uteri. It increased, and an operation was proposed, but M. Dupuytren discountenanced the idea.

*Case.* A female applied to M. Dupuytren, with violent pains in the rectum. He passed the finger into the gut, and found a tumor of a conical figure external to its anterior wall; the latter not being adherent to it. The finger in the vagina at first discovered nothing, but, at length, the tumor was found to proceed from the posterior surface of the cervix uteri, and to pass between the vaginal and rectal parietes.

*Case.* Another patient had a tumor on the anterior and lateral surface of the cervix. It pressed on the bas-fond of the bladder, occasioning much distress, and a frequent desire to make water.

*Case.* And another female had the tumor placed between the cervix uteri, the bas-fond of the bladder, and the posterior surface of the urethra. She had frequent desire to make water, in consequence of the pressure on the bladder, yet suffered from retention, the effect of compression of the urethra.

With respect to the prognosis, we see nothing in either of the works before us which demands a special notice. The experience of Dugés and Boivin, on one point, is worth recording.

"There are numerous cases which prove that sterility is not an inevitable result of this disease: we, with others, have had occasion, however, to observe that pregnancy may not proceed to its usual period; and abortion, in this case, has been sometimes serious, and even fatal. In other cases no serious result has occurred beyond that of common abortion. The same remarks may be made respecting pregnancy proceeding to its full time; delivery may take place naturally, though there are instances in which hæmorrhagy, or other consequences, have proved serious, or even fatal." 209.

The consideration of the symptoms will render much reference to the remarks of the authors on diagnosis needless. The only point which we think it advisable to notice is the mode, and sometimes the difficulty, of distinguishing between polypus and inversion of the uterus. We shall venture to extract the observations of Dugés and Boivin upon this head. They are lengthened, but useful.

"But the affection most liable to mislead the practitioner is that of introversio uteri. Levret says, in his memoir (art. 1er), that it is necessary to read the curious cases in which polypi have been removed by the ligature or excision, and mistaken for the inverted uterus. We have already spoken of hollow polypi, which have particularly occasioned this confusion, and we have also said (inversion) that some practitioners have been so far deceived in this respect, as to maintain that the uterus had been extirpated, although the catamenia had re-appeared afterwards, and the patient had even become pregnant. We have also observed that, in some cases, the ligature or knife has been injudiciously applied to the uterus, from an impression that the tumor was polypus. There are several symptoms and circumstances discoverable on examination, which, in fact, apply to both these affections; such as hæmorrhagy, mucous discharges, draggings, the form and situation of the tumor: the sources of their diagnosis are as follow.

We must first notice the events which marked the origin of inversion at the very moment of delivery, and which we have detailed in one of the preceding chapters. If, at this early stage of inversion, in the first degree, the existence of polypus be suspected, it may easily be ascertained, on external examination, that the rounded tumor, felt by the finger in the os uteri, exactly corresponds with a similar depression of the fundus of the uterus; and simple reduction would, in most cases, remove all doubt. But when the affection has been of longer continuance, and the tumor has passed through the os uteri into the upper part of the vagina, the diagnosis may present greater difficulties: it will nevertheless be right to consider—1, that it is only in cases in which the polypus has descended almost to the os externum, that the fundus uteri has been so far depressed as to be no longer felt upon examination by the hypogastrium, and may even then be reached by forcing back the polypus into the vagina: 2, that the fundus uteri is of a rounded form; or, if depressed, not proportionably so with the sinking of the tumor in the pelvis; 3, that this latter tumor, being fairly examined between the fingers placed above the pubes and those introduced into the vagina, is much larger (twice as large at least) than the uterus, as ascertained at the same time. Leaving these conjectures, however, it might be ascertained, by the finger or the elastic catheter, that the inverted uterus is encircled at its neck with a cul-de-sac of little or no depth; whilst, in a case of polypus, the sound will be carried very far along its pedicle, beyond the os uteri through which it had passed. We will just add that the consistence of the uterus is usually softer, allowing it to become rugous and furrowed longitudinally, in consequence of the cavity in its centre;—remarks which may therefore apply to the hollow polypus. These polypi, indeed, generally filled with fluid are liable to evacuate their contents at intervals by some fistulous orifice, frequently inducing considerable changes in their form and consistence. We shall not dwell upon the deep colour and ecchymoses of the uterus, because polypi also present numerous appearances of this kind; but we may observe, with Levret and others, that the uterus is painful and tender to the touch, while polypus, when touched, scratched, or pricked, remains insensible.

A voluminous polypus, protruding from the os externum, may easily be distinguished from inversion of the last degree; its pedicle is found to be contracted and solid, and encircled to some depth by the vagina; while the base of the inverted uterus is large, soft, containing a portion of the rectum and of the bladder, together with the matter collected in those organs. This base, constituting the parietes of the vagina, itself inverted, is continuous with the borders of the os externum, and the finger cannot be pressed deeply into any point of its contour." 206.

M. Dupuytren passes successively in review cauterization, torsion, crushing, tearing away, and ligature, as modes of removing polypi. But these may be disregarded, for the purpose of ascertaining the practice preferred and adopted by himself. It is excision.

In order to apply, or rather to shew how M. Dupuytren applies this, the reporters retrace their steps, and occupy themselves again in distinguishing the various species of these tumors. Yet we think that the tedious recapitulation may be spared. We shall suppose that the polypus is contained in the cavity of the uterus, and that the finger is capable of ascertaining its existence there. The question arises—what are the indications for an operation? what the period for its performance?

Those questions may receive the general reply—that urgent symptoms can alone demand, or even justify it. Profuse and exhausting bloody discharges, commencing degeneration of the tumor, and sufferings that seriously impair the health, are the usual signs indicative of its propriety.

We will imagine that these or other circumstances call for interference on the surgeon's part. The mode of operating must occupy his attention. The state of the os uteri must, of course, be ascertained. If sufficiently dilated, M. Dupuytren approves of the exhibition of the ergot of rye, provided there is reason to believe that the polypus is of the pediculated kind.

*Case.* M. Guersent, jun. was called to see a female, who was suffering from pains in the abdomen, groins, uterus, &c. accompanied with hæmorrhage from the vagina. On examination, he found the os uteri dilated, and distinguished in the uterus a round smooth body, which he readily ascertained to be a fibrous tumor. Thinking that the os uteri would permit the passage of the tumor through it, he prescribed the ergot of rye. The uterus contracted, and propelled the polypus into the vagina, where it was conveniently placed for an operation.

If the os uteri is little or not at all dilated, mechanical dilatation has been recommended and employed. M. Dupuytren relates a case in which it was adopted.

*Case.* Some years ago, M. Dupuytren was summoned in great haste to a lady, who was almost in extremis. He learnt that she had been labouring under polypus of the uterus, that it had presented at the cervix, that the os uteri had not enlarged sufficiently to permit the passage of the polypus, and that attempts had in consequence been made to dilate it by means of sponges and prepared gentian root. These measures had given rise to intense peritonitis, for which M. Dupuytren's attendance was demanded.

This case, and considerations which it illustrates and strengthens, induce M. Dupuytren to discountenance altogether mechanical dilatation of the os uteri.

The ergot of rye has been recommended where the os uteri is sufficiently open. Yet it frequently will fail, for the cervix is not, as in pregnancy, softened and attenuated, and gradually stretched. In cases of polypus it preserves its thickness, nay, chronic inflammation may augment the latter, and its great rigidity offers an invincible obstacle to success. When this is the case, M. Dupuytren divides the cervix by incision, a method which he has frequently adopted with success. After this has been done, the ergot acts in a satisfactory manner.

Division of the cervix may be effected in two ways:—by division from within outwards with the probe-pointed bistoury or bistoire cachée: or, by division from without inwards, with the sharp-pointed bistoury thrust through the cervix. M. Dupuytren assures us that this operation has proved in his hands the means of cure, in cases considered beyond the reach of art.

After incision of the neck and dilatation of the os uteri, the hooked forceps of Museux should be applied to the tumor, and the latter drawn down by its assistance. In many cases this is impossible, at least to an adequate extent. M. Dupuytren advises us, under these circumstances, to drag the uterus to the lower outlet, and effect its "demi-renversement." If this again is unattainable, the cervix should be incised more freely, and the tumor cut out from the uterine cavity. The indications are the same when the pedicle of a polypus which has passed into the vagina is so closely encircled by the cervix that it is not possible to divide it above the os tincæ. Such are the recommendations, such apparently the practice of M. Dupuytren. Yet the former would appear to the cautious English surgeon to be injudicious—the latter to be hazardous and rash. The following case was notwithstanding a successful one.

*Case.* A milliner, æt 49, was received in the Hôtel Dieu in December, 1823, on account of a large polypus which projected between the labia. It was red, hard, and ulcerated in several situations. The finger introduced into the vagina could be easily passed round the pedicle of the tumor, which was forcibly grasped by the cervix of the uterus. M. Dupuytren removed the polypus on the 13th of the month. He seized and dragged it out of the vagina by the hooked forceps of Museux, applying fresh as the tumor descended lower and lower till the neck of the uterus appeared at the vulva. The pedicle was then found to be grasped by it too tightly to permit the section above it. M. Dupuytren, therefore, divided the cervix from within outwards with the probe-pointed bistoury, and then, with curved scissors, cut through the pedicle within the uterus. No hæmorrhage succeeded, the uterus reascended into the vagina, and the patient recovered without the supervention of symptoms of material consequence.

When the polypus has descended into the vagina, the operation of excision is in many cases simple. But the following circumstances may occasion difficulties.

1. Sometimes the uterus is depressed with difficulty, or the polypus from other causes refuses to descend, and its pedicle cannot be brought out at the vulva. M. Dupuytren, in such a case, recommends the surgeon to divide the pedicle at its most contracted part with a bistoury, the blade of which is partially encircled by a bandage, or with proper curved scissors, the instrument being carefully guided on the fingers of the left hand in the vagina. Such a proceeding should be likewise had recourse to, if the pedicle appears incapable of sustaining the necessary traction.

2. Before the surgeon attempts to drag the polypus down, he should ascertain if it adheres to the surrounding parts. If adhesions exist, they should be carefully divided with long and strong scissors curved on the blade-plane, and with blunt edges to tear through the adhesions and the vessels they contain, rather than to cut them. The dissection is delicate, and should be carefully performed.

3. A circumstance usually accompanies the exit of the polypus from the vagina, which should be mentioned and remembered. It is this. A jet of blood takes place, as occurs in delivery, and after the abstraction of fibrous polypi from the nose. It arises no doubt from some of the vessels of the vagina. It is momentary and of little consequence.

4. Polypi are occasionally too large to admit of being drawn through the vulva. If the difficulty arises from the narrowness of that aperture, M. Dupuytren does not hesitate to divide its posterior commissure—if it depends on the magnitude of the tumour materially exceeding that of the lower pelvic out-let, the former must be crushed, or torn, or cut into fragments with the bistoury.

The preference displayed by M. Dupuytren for the knife, is evidence in itself that he does not dread and has not often met with hæmorrhage. Yet cases may occur in which, from the pulsation of or on the tumor, it might seem to contain arteries of considerable magnitude. In such cases he would recommend the application of a ligature prior to the division of the pedicle. But he does not recollect that he ever had occasion to resort to this precaution.

The polypus without a pedicle, imbedded in the tissue of the uterus, *may* require or justify an operation. *If* it does, M. Dupuytren proceeds in this manner :—he first makes a semi-elliptical incision round the anterior half of the tumor at its base. As soon as the edges of the wound retract, and the tumor becomes prominent, a similar incision is made posteriorly, the two, of course, embracing the morbid growth. The edges of both wounds retracting expose the origin of the tumor, which if seated in the cellular tissue below the mucous membrane may be turned out with the finger or the handle of the scalpel: and if placed in the inter-fibrous cellular tissue may require a few touches of the bistoury.

M. Dupuytren has sometimes met with polypi displaying an enlargement in the vagina, and one also in the uterus to which the latter is attached by a pedicle. Such polypi will frequently come under the class of those which cannot clear the os uteri, and will require the same treatment. For this we refer to a former part of the article.

The management of the patient after the operation is usually simple. As soon as the pedicle is cut the uterus rises suddenly to its proper situation in the pelvis, and the bleeding which ensues is almost always moderate and of brief duration. The discharges which existed previously immediately cease, and unless the patient was irretrievably exhausted a few days commonly restore her to health. Yet management and caution are required for a longer period, a dangerous inflammation of the uterus and peritoneum being sometimes insidiously developed. We saw a patient die with pus in the veins of the uterus, and inflammation of the peritoneum and the pelvis after the application of the ligature.

We must now conclude this article. On the whole, it may be regarded as a fair exposition of the principal fact and opinions contained in the lecture of M. Dupuytren. That lecture occupies upwards of 160 pages. Its compression into 14 is no trivial operation, facilitated though it is by the natural turgescence, diffuseness, and repetitions of French writings.

